# A Flexible Asymmetric Supercapacitor with High‐Performance and Long‐Lifetime: Fabrication of Nanoworm‐Like‐Structured Electrodes Based on Polypyrrole‐Thiosemicarbazone Complex

**DOI:** 10.1002/smtd.202401140

**Published:** 2024-10-30

**Authors:** Elif Avcu Altıparmak, Sibel Yazar, Tulay Bal‐Demirci

**Affiliations:** ^1^ Department of Chemistry Engineering Faculty Inorganic Chemistry Department Istanbul University‐Cerrahpasa Istanbul 34320 Turkey; ^2^ Department of Chemistry Engineering Faculty Department of Physical Chemistry Istanbul University‐Cerrahpasa Istanbul 34320 Turkey

**Keywords:** energy storage, polypyrrole, pseudocapacitor, supercapacitor, thiosemicarbazone, transition metal complex

## Abstract

A thiosemicarbazone‐based iron(III) complex is prepared and used in the preparation of a supercapacitor electrode material. This electrode is produced by a solvothermal reaction of polypyrrole and the complex on carbon felt. The characterization of the complex and material is carried out using UV‐vis, elemental analysis, FT‐IR, XRD, BET, and TGA methods, and the surface morphology is examined using SEM technique. Because the interaction of electrode and electrolyte is of great importance in energy storage systems, as the surface area and pore volume increase, electrode ions at the electrode/electrolyte interface leak to the inner surfaces and interact with the larger surface area, which increases the charge storage performance. The electrode material, nano‐worm structure, reached the highest specific capacitance value of 764.6 F g^−1^ at 5 mV s^−1^. Compared to the capacitance value of polypyrrole in its pure form, it is observed to exhibit an 187.2% increase. The highest specific capacitance value of the asymmetric supercapacitor (ASC) formed with a graphite electrode is 318.1 F g^−1^ at the current density of 1 Ag^−1^. Moreover, ASC reached a wide working potential of 1.8 V in an aqueous electrolyte and exhibited ultra‐long cycle life (112%), maintaining its stability after 10 000 cycles.

## Graphical abstract

An asymmetric supercapacitor including thiosemicarbazone exhibited a large operating potential of 1.8 V, allowing it to reach higher energy density values (56.5 Wh kg^−1^ at 1.0 Ag^−1^) and is stable up to 10 000 cycles.

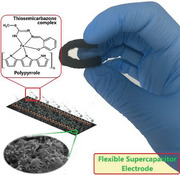

## 1 Introduction

Fossil resources have been widely used for the past centuries. This has seriously threatened human and environmental security and has caused global climate warming with the greenhouse effect. These harmful effects will continue unless the unconscious consumption of energy resources is prevented, and the available energy cannot be stored efficiently. To minimize these negative effects, research is being conducted on environmental, sustainable, and highly reliable energy storage systems.^[^ ^1^, ^2^ ^]^ Supercapacitors, known as ultracapacitors among these units, have been one of the intensive research topics due to their fast charge‐discharge behaviors, long cycling life, high power, and high energy.^[^ ^3^, ^4^ ^]^ Based on their charge storage characteristics, there are two types of supercapacitors. The first type is an electrochemical double layer capacitor (EDCL) that stores electrical energy by stratifying charges at the electrode‐electrolyte interface through electrostatic interaction. This physical phenomenon results in fast charge–discharge profiles, high power density, and long cycling life.^[^ ^5^ ^]^ Carbon‐based substances such as graphite, graphene, and carbon nanotubes are generally used in the electrode material where the charges are stored.^[^ ^6^, ^7^ ^]^ The other type of supercapacitor uses redox reactions to store electrical energy and is called a pseudocapacitor. Metal oxides‐hydroxides‐nitrides and conductive polymers are among the well‐known pseudocapacitive materials.^[^ ^8^, ^9^, ^10^, ^11^ ^]^ The energy density of these supercapacitors is higher compared to EDLC types, but the long life cycle of the materials providing storage is limited by the occurrence of chemical reactions.^[^ ^12^ ^]^ Polypyrrole (PPy) is a π‐electron conjugated conducting polymer preferred as an energy storage material due to its excellent electrical conductivity, high environmental stability, and less toxicological properties.^[^ ^13^, ^14^, ^15^, ^16^, ^17^ ^]^ A dopant can be added during PPy polymerization to enhance the active performance of PPy‐based supercapacitor electrodes. It can be ensured that the polymer obtained by adding the dopant gains superior structural and morphological properties.^[^ ^18^, ^19^ ^]^

Thiosemicarbazones are a kind of Schiff bases, and these compounds are obtained by condensation of a thiosemicarbazide molecule with carbonyl groups.^[^ ^20^ ^]^ Thiosemicarbazones have become an important family of −N, −S donor ligands since their discovery due to the variety of their structure, variable donor properties, and biological applications.^[^ ^21^, ^22^, ^23^, ^24^ ^]^ Thiosemicarbazone derivatives have potent coordination affinity, intense selectivity, and stability against many metal ions.^[^ ^25^ ^]^ The electrochemical behavior of thiosemicarbazones and their complexes are associated with their ability to participate in redox reactions.^[^ ^26^, ^27^, ^28^ ^]^ These reactions involve the transfer of electrons between the compound and an electrode surface. The behavior of thiosemicarbazones during these reactions can provide valuable information regarding their potential applications in various electrochemical processes.^[^ ^29^, ^30^ ^]^ The redox potentials of complexes come from extensive electron delocalization in the thiosemicarbazone moiety and oxidation and reduction of the central metal ion and ligand.^[^ ^31^, ^32^ ^]^ These properties allow them to be used as antioxidants and catalysts while also making them potentially interesting for supercapacitor applications.^[^ ^28^, ^33^ ^]^ The redox activity of the metal center can potentially contribute to the energy storage capabilities of supercapacitors.^[^ ^26^ ^]^ These complexes undergo reversible redox reactions, enabling efficient charge storage and rapid charge/discharge cycles.^[^ ^34^ ^]^ Furthermore, iron (III) complexes of thiosemicarbazones have been investigated as redox‐active materials.

The redox behavior of iron such as in Fenton‐type reactions is a significant characteristic that determines its participation in electron transfer processes and its utility in various biological, chemical, electrochemical, and industrial applications.^[^ ^35^ ^]^ The redox activity of iron (III) complexes allows for the reversible storage and release of charge. These properties make iron (III) thiosemicarbazone complexes valuable in various electrochemical applications, such as energy storage systems.

Carbon felt, due to its high electrical conductivity, and low cost, was selected in preparation for a supercapacitor as a current collector. Carbon felt was functionalized, to C─O, C═O, C─O─C groups for interaction with polypyrrole were generated, and positively charged polypyrrole was chemisorbed onto functionalized carbon felt (C/PPy). Polypyrrole (PPy) is a π‐electron conjugated conducting polymer and was preferred as an energy storage material because of its excellent electrical conductivity, high environmental stability, and less toxicological properties.^[^ ^36^ ^]^ So, the electro‐chemically active sites on the surface of the electrode were increased.

Arvas et al. reported the synthesis of polypyrrole (PPy) based electrodes doped with thiosemicarbazide derivatives (TSC, N‐Phenyl TSC, N‐Cyclohexyl TSC) and their electrochemical performance in supercapacitor field. The PPy/N‐Cyclohexyl TSC (2.5 mm) electrode was reported to exhibit a capacitance of 300.5 F g^−1^ and a current density of 0.5 A g^−1^ over a potential operating range of 1.1 V in 0.5 m H_2_SO_4_ electrolyte. The symmetrical supercapacitor device obtained using PPy/N‐Cyclohexyl TSC (2.5 mm) electrode was reported to exhibit 90.4% and 81.5% capacitance retention rate in 0.5 m H_2_SO_4_ and 1.0 m Na_2_SO_4_ electrolytes and at the end of 5000 cycles life cycle test, respectively.^[^ ^33^ ^]^ Hong et al. fabricated polystyrene@polypyrrole (PS@PPy) core‐shell nanoparticles and studied the removal of PS cores using organic solvents to form hollow PPy nanospheres. They used six different organic solvents (cyclohexane, toluene, tetrahydrofuran, chloroform, acetone, and N,N‐dimethylformamide (DMF)) to etch PS cores. PPy nanospheres synthesized with DMF were reported to exhibit superior electrochemical properties. This was attributed to high effective PS removal efficiency, increased specific surface area, and improved charge transport efficiency. The specific capacitance of PPy nanospheres was reported to be 350 F g^−1^ at 1 A g^−1^.^[^ ^37^ ^]^ MacDonald et al. examined the SrFe_12_O_19_ (SFO) material and its composites with polypyrrole (PPy). An organic surfactant‐charge transfer agent and high‐energy ball milling (HEBM) were utilized to fabricate the composite electrodes. The impact of HEBM and SFO content on capacitive characteristics was examined in the composites. Magnetic measurements demonstrated the influence of HEBM on magnetic properties, with the composites exhibiting favorable magnetic characteristics and improved capacitive qualities.^[^ ^38^ ^]^

Here, the synthesis of polypyrrole with a thiosemicarbazone‐derived iron(III) complex using a 2‐hydroxy‐5‐bromo benzaldehyde‐S‐methyl isothiosemicarbazone (PPy/TSC) complex has revealed enhanced surface properties of polypyrrole exhibiting a unique nanoworm‐like morphology that has not been reported in previous studies. Thiosemicarbazone as a nitrogen‐rich dopant was added to improve the supercapacitor electrode active site and to enhance the performance of PPy‐based materials. The exceptional long‐term durability of PPy/TSC supercapacitors is evidenced by their ability to maintain capacitance with a slight increase of up to 112% after 10 000 cycles in a cost‐effective aqueous electrolyte such as 3.0 m NaCl. The synthesized supercapacitor electrode represents a remarkable advance in the investigation of conducting polymers, which often exhibit a limited lifetime in practical electronic applications.

## 2 Experimental Section

### 2.1 Chemicals

All chemicals were used directly from commercial sources without further purification and were of ACS reagent quality. The solvents were HPLC purity and ultrapure for water.

### 2.2 Preparation of PPy‐TSC Complex Electrode Material

To yield PPy‐TSC complex electrode material, PPy, and TSC Complex were prepared separately. Afterward, a composite material (PPy‐TSC complex electrode) was formed by combining the PPy and TSC Complex on the carbon felt in an autoclave.

### 2.3 Preparation of Electrodes

Electrodes were washed with acetone for 30 min in an ultrasonic bath to remove contaminations on the structure surface and dried at room temperature. Then, they were treated with HNO_3_ and stored for 72 h at room temperature. The oxidized components were rinsed with deionized water until the pH became neutral. After that, the electrodes dried in an oven at 40 °C for 12 h.

### 2.4 Preparation of PPy Electrode Material

PPy electrode material was prepared by solvothermal reaction of pyrrole (Py) and FeCl_3_.6H_2_0 in an autoclave. First, the mixture of 0.3 g Py and 0.54 g FeCl_3_.6H_2_O in ethanol: water (1:1) solution was stirred for 30 min at room temperature. Then, the resulting mixture was transferred to an autoclave containing an electrode in it. After that, the autoclave was kept in an oven at 180 °C for 12 h. Finally, the obtained composite electrodes were washed in a mixture of ethanol: water (v:v, 1:1) and dried under vacuum at 90 °C for 6 h.

### 2.5 Preparation of TSC Complex

**Figure 1:**
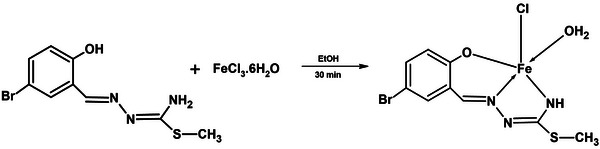
Synthesis of the TSC complex.

The 5‐bromo‐2‐hydroxy‐benzaldehyde‐S‐methyl isothiosemicarbazone was prepared by lit method.^[^ ^39^, ^40^ ^]^ 5‐bromo‐2‐hydroxy‐benzaldehyde‐S‐methyl isothiosemicarbazone (1 mmol, 0.29 g) was dissolved in ethanol in a reaction flask, and FeCl_3_.6H_2_O (1 mmol, 0.27 g) was added to the reaction medium. After 30 min of stirring, the black solution was formed. Evaporation of the solution yielded a black powder product (**Figure** **1**).

### 2.6 Characterization Data of the TSC Complex

The Color: Black; Yield: 90%; m.p. (°C): >350; molar conductance (in DMSO at 25 °C): 11.5 Ω^−1^cm^2^ mol^−1^, Elementel Analysis: *Anal*. Calc. For C_11_H_14_BrClFeN_3_O_2_S (3954 g mol^−1^): C, 27.33; H, 2.55; N, 10.63; S, 8.11; Found: C, 27.31; H, 2.54; N, 10.64; S, 8.12%. IR (cm^−1^): υ(─OH) 3390; δ(N─H) 1637; υ(C═N) 1592, 1574; υ(C─O) 1155; υ(C─S) 702. UV–vis (10^−5^ m, CHCl_3_): ((λ (ε)) 430.5 (7080), 299.5 (19900), 236.5 (18900), 204.5 (14100). *m*/*z* +c HRMS, (relative abundance%): 289.9 (100%) [L+2H]^+^, 287.9 (94%) [L]^+^, 286.9 (29.8%) [L‐H]^+^, 632.8 (61.5%) [(M‐Cl)+L]^+^, 378.3 (25.5%) [[(M‐Cl)+H_2_O]^+^.

### 2.7 Preparation of PPy‐TSC Complex Electrode Material

**Figure 2:**
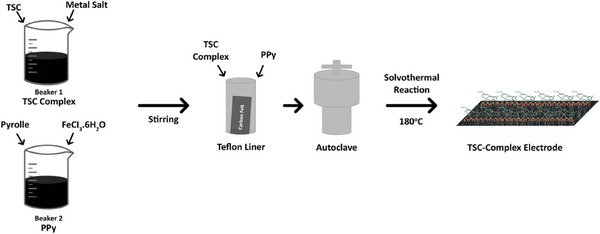
Scheme of the PPy‐TSC complex molecular structure.

The resulting mixtures of TSC Complex and PPy prepared as described above were transferred to an autoclave containing an electrode. The autoclave was subsequently stored in a 180 °C oven for 12 h. Concentration optimization of TSC Complex was investigated between 0.01 and 0.05 m. Finally, the obtained composite electrodes were washed in ethanol: water (v:v, 50:50) mixture and dried under vacuum at 90 °C for 6 h (**Figure** **2**).

### 2.8 Structural and Chemical Characterization of the Electrodes

The surface characteristics of the obtained electrodes were investigated by using a scanning electron microscope (Philips XL 30 SFEG SEM). Thermogravimetric analyses (TGA) of the electrodes were carried out using the SII 6000 Exstar TG/DTA 6300 device, and it was conducted to examine the thermal stability of the sample in the presence of nitrogen (N_2_) gas. BET analysis was obtained using Quantachrome Quadrasorb SI Brunauer–Emmett–Teller (BET) device after 24 h of degassing at 120 °C. The FT‐IR spectra of electrodes were obtained with the Agilent Cary 630 FTIR spectrometer with a Diamond ATR from 4000 to 600 cm^−1^. The powder X‐ray diffraction (XRD) patterns of PPy and PPy‐TSC Complex were determined with the PANalytical X'Pert PRO XRD device (Cu, 45 kV–40 mA and CuK1.54059 Angstrom).

### 2.9 Electrochemical Analysis of the Electrode Materials and Supercapacitor

An Ivium Vertex Instruments Potentiostat/Galvanostat (Ivium Technologies B.V, Netherlands) was used for electrochemical measurements. Cyclic voltammetry (CV), galvanostatic charge/discharge (GCD), and electrochemical impedance spectroscopy (EIS) methods were used.

The capacitance performances of the electrodes were tested with a 3‐electrode combination using Pt wire as a counter electrode and Ag/AgCl as a reference electrode in the 3.0 m KCl aqueous electrolyte at ambient temperature. Cyclic voltammograms were recorded between −0.25 and 0.4 V. EIS analysis was conducted in the frequency range of 0.1 Hz to 100 kHz with an AC amplitude of 0.01 V in 3.0 m KCl solution. Measurements were performed in a two‐electrode system for an asymmetric supercapacitor with dimensions of 1 × 1 cm^2^ using the PPy‐TSC complex working electrode and graphite sheet. The 3.0 m KCl solution was used as an electrolyte. The specific capacitance was calculated from the CV curves by Equation (1). Also, based on the GDC measurements, the specific capacitance can be determined with the help of Equation (2) presented below:^[^ ^4^ ^]^

(1) $$\mathrm{Cs}=\frac{\oint_{V1}^{V2}I\;dV}{m\times v\times \Delta V}$$

(2) $$\mathrm{Cs}=\frac{I\times \Delta t}{m\;\times \Delta V}$$

where Cs (Fg^−1^) is the specific capacitance, $\oint_{V1}^{V2}I\;dV$ is the integrated field from the CV measurement, ΔV (V) is the working potential window, m(g): is the amount of active substance in the electrode, and v(mV s^−1^) refers to the scan rate, I(A) is the discharge current and Δt(s) is the discharge time.^[^ ^41^ ^]^

The specific energy density E (Wh kg^−1^) and specific power density P (W kg^−1^) were calculated with the given Equations (3) and (4).^[^ ^42^ ^]^

(3) $$E=\frac{\;Cs\;\times {\left(\Delta V\right)}^{2}}{2\;\times 3.6}$$

(4) $$P=\frac{3600\times E}{\Delta \;t}$$

## 3 Results and Discussion

The TSC Complex in black powder form was produced by the reaction of 2‐hydroxy‐5‐bromobenzaldehyde‐S‐methyl‐isothiosemicarbazone and FeCl_3_ and was soluble in alcohol, chloroform, DMF, and DMSO but not soluble in water. The molar conductivity of the complex was measured in a 10⁻^3^ m solution of DMSO, yielding a value of 11.5 S cm^2^ mol^−^¹. This value indicates a non‐ionic structure for the complex, although the relatively higher conductivity than expected is attributed to the presence of chloride ions coordinated to the iron(III) center.^[^ ^43^ ^]^

The UV–vis spectrum of the compound was recorded in chloroform at a concentration of 10⁻⁵ m (Figure S4, Supporting Information). In The UV–vis spectrum of the complex, the observed absorption peaks at 204.5, 236.5, 299.5, and 430.5 nm can be assigned to π → π*, n → π* and charge‐transfer transitions. In the IR spectrum of the complex, the bending band of the —NH group was seen at 1637 cm^−1^. No band was clearly seen because of the enlargement between 3500 and 2700 cm^−1^ caused by the —OH group related to H_2_O in the molecule structure.^[^ ^47^ ^]^ The C═N bands belonging to the imine group in the thiosemicarbazone structure were recorded at 1592 and 1574 cm^−1^. In the ESI‐MS spectrum of the TSC complex, peaks corresponding to the fragments [L+2H]^+^, [L]^+^, and [L‐H]^+^, which represent the molecular weight of the ligand, were recorded with relative abundances of 100% (289.9), 94% (287.9), and 29.8% (286.9), respectively. The secondary dominant peak in the spectrum, observed at m/z 632.8 with a relative abundance of 61.5%, was attributed to the structure of [(M‐Cl)+L]^+^, resulting from the interaction of 2 moles of the ligand with 1 mole of the metal ion. The molecular peak of the complex [(M‐Cl)+H_2_O]^+^ was recorded at m/z 378.3 with a relative abundance of 25.5%.

The values obtained from the elemental analysis of the complex were consistent with the molecular formula.

**Scheme 1:**
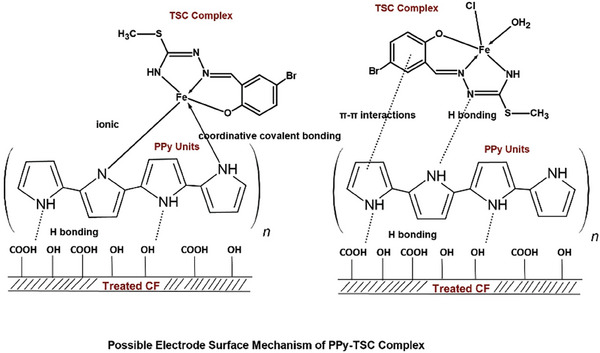
Possible electrode surface mechanism of PPy‐TSC Complex on carbon felt.

The solvothermal reaction of the complex with polypyrrole was carried out on a carbon felt and a pseudo‐electrode was fabricated (PPy‐TSC Complex). **Scheme** **1** presents the proposed mechanisms for the interaction between PPy groups on the electrode surface and the TSC complex. Structural characterization primarily indicates that a metal complex has been obtained, with the metal ion bound to the thiosemicarbazone ligand. This metal ion can form ionic or coordinative covalent bonds with the ‐NH groups present in the polypyrrole units. Additionally, hydrogen bonds may form between the NH groups in the structure of the TSC complex compound and the polypyrrole. Additionally, π–π interactions may be observed between the phenolic unit in the thiosemicarbazone molecule and the polypyrrole.

**Figure 3:**
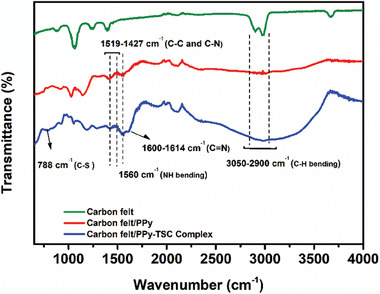
FT‐IR Spectra of the electrodes.

Comparison of pure carbon felt, C‐PPy and C‐PPy‐TSC Complex electrodes was followed by IR spectra (**Figure** **3**). On analyzing the FTIR spectrum of the PPy, the band ≈3400 cm^−1^ represented the N─H stretching of the pyrrole and this wide band supported the polymerization. The peak observed at 1560 cm^−1^ was assigned to the ─NH bending and indicated the presence of pyrrole in the structure. The peaks at 1519 and 1427 cm^−1^ corresponded to the symmetric and asymmetric stretching modes of C─C and C─N on the pyrrole ring.^[^ ^44^ ^]^ Peaks of aromatic C─H stretching in the range of 3050–2900 cm^−1^ were intensely observed due to polymerization.^[^ ^45^ ^]^ When compared with the spectrum of pure CFt, it is clearly seen that the polypyrrole is bound to the structure. In addition, when carbon felt was functionalized for interaction with polypyrrole, the generated groups of C═O, C─O, C─O─C were observed at 1635, 1364, and 1155–1050. In the FTIR spectrum of the PPy‐TSC complex electrode, the C═N groups at 1614 and 1600 cm^−1^, and the —NH group at 1653 cm^−1^ related to the TSC complex were clearly seen.^[^ ^39^, ^46^ ^]^ While these peaks were formed, the typical peaks of PPy at 1560 and 1519 cm^−1^ were preserved.

The further expansion of the 3300 cm^−1^ region supported that the polymerization was enhanced as a result of the contribution of the thiosemicarbazone complex to the structure. The peak at 788 cm^−1^ was attributed to the C─S stretching of the ─S─CH_3_ group in the thiosemicarbazone molecule. The data of the spectrum showed that the thiosemicarbazone complex bonded to the composite electrode structure and enhanced PPy polymerization.

**Figure 4:**
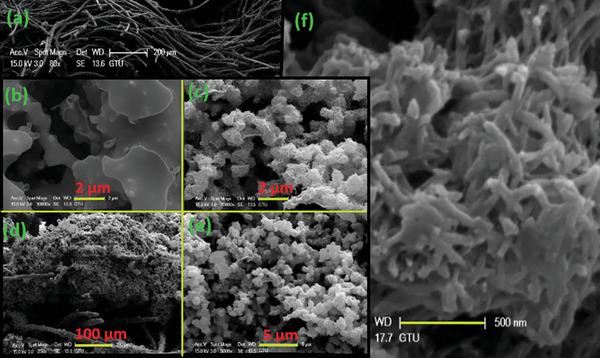
SEM Images of a) carbon felt, b) PPy, and c–f) PPy‐TSC Complex at different magnifications.

The SEM images of carbon felt (a), PPy (b), and PPy‐TSC Complex (c–f) were investigated (**Figure** **4**). It was observed that the pure carbon felt had a flat surface, ≈10 µm wide (Figure 4a). The PPy electrode structure obtained by the polymerization of pyrrole exhibited a more irregular, cauliflower‐like structure compared to the pure carbon felt^[^ ^48^ ^]^ (Figure 4b). This structure indicated that polypyrrole adsorbed onto the carbon felt surface and was stabilized through π–π interactions between the carbon and oxygen atoms of carbon felt and the nitrogen atoms of the PPy electrode. The SEM image of the TSC complex is presented in Figure S1 (Supporting Information). It was observed that it exhibited a surface consisting of small clusters but not continuous compared to polymeric materials. Figure 4c–f revealed a nano‐worm morphology, confirming the formation of the PPy‐TSC complex electrode structure. This new morphology significantly increased the surface area of the electrode. This electrode structure was formed by the interaction of the TSC complex with iron(III) ions bonded to the PPy electrode through ionic and coordinative covalent bonds. The surface structure of the electrode plays an important role in the electrochemical energy storage materials. As a result, the electrochemical capacitance properties of the nano‐worm structure PPy‐TSC complex material reached higher values compared to the PPy structure. Because the interaction between the electrode and electrolyte is of great importance in energy storage systems, as the surface area and pore volume increase, electrode ions at the electrode/electrolyte interface leak to the inner surfaces and interact with the larger surface area, which increases the charge storage performance.

In addition, previous investigations have indicated that electrically conductive polypyrrole films, produced through diverse manufacturing techniques, exhibit variations in molecular anisotropy, electrical conductivity, and surface characteristics. The PPy polymer chain can be elongated in one direction, exhibiting morphological anisotropy throughout its thickness. It flexes and inverts in a consistent manner throughout a redox cycle. The mobilization characteristics of the anisotropic PPy film are significantly influenced by the cation size in the electrolyte solution, with reports indicating that its bending at room temperature occurs more slowly for larger cations.^[^ ^49^, ^50^ ^]^ Figure 4 illustrates the development of polypyrrole films exhibiting various morphologies. The polypyrrole films synthesized with the addition of the TSC complex to the medium displayed a structurally fringed morphology.

**Figure 5:**
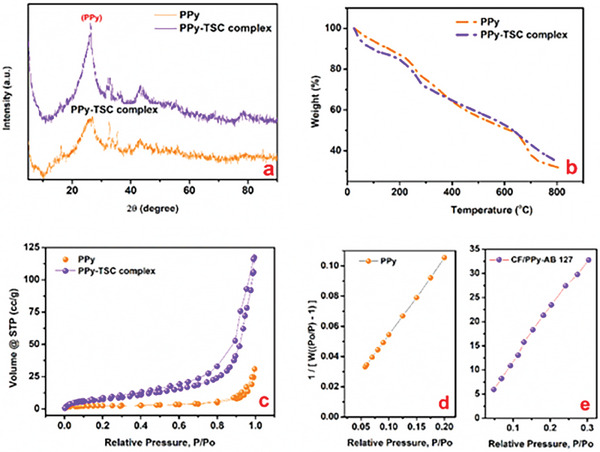
a) XRD pattern, b)TGA analysis, c) N_2_ adsorption‐desorption isotherms, d,e) BET surface area plot of PPy and PPy‐TSC complex coated on carbon felt electrode.

**Figure** **5**a shows the spectra of XRD analysis of the PPy and PPy‐TSC complex. The ambiguous structure of the XRD graphs clearly showed the amorphous characteristics of the polymers The XRD pattern of the PPy‐TSC complex exhibited four diffraction peaks at 2θ = 26°, 32°, 33°, and 43°. It is known that the XRD pattern points to a hexagonal graphitic structure^[^ ^51^ ^]^ The peak at 26° indicated that polypyrrole was successfully bonded to the electrode surface.^[^ ^52^ ^]^ The intensity of the polypyrrole peak increased and sharpened in the structure bonded to the TSC complex. This sharpness indicated that the compound bonded to the structure had a favorable effect on the polymerization of polypyrrole. Upon binding of the TSC complex to PPy, the peaks at 33° and 35° shifted to 32° and 33°, respectively, and these peaks were attributed to the ambient Fe(III) ion.^[^ ^53^ ^]^ The peak at ≈2θ ≈ 43° is a distinctive feature associated with the crystal plane of low‐grade carbon in the carbon felt, and its intensity was also observed to increase.^[^ ^54^, ^55^ ^]^ The high intensities of the peaks indicated that the crystallinity of the PPy‐TSC complex electrode was higher than that of the PPy electrode. It was observed that the added TSC complex improved the chain bond structure of the material.

TGA thermal analysis studied PPy and PPy‐TSC complex electrodes between 0 and 800 °C to determine the thermal stability and structure estimation. The thermogram of PPy and PPy‐TSC complex electrodes is shown in Figure 5b. The first decomposition step corresponded to the removal of water molecules from the structure within the temperature range of 25–150 °C. The next step took place in the temperature range 150–350 °C, which shows the dopant molecules removed from the PPy chains in the thermogram of PPy and the weight change was 11.18%.^[^ ^48^ ^]^ The more pronounced sloping decline noted in the PPy‐TSC complex relative to PPy in the second stage might also be ascribed to the molecular dissociation of TSC.^[^ ^56^ ^]^ (The weight change for the PPy‐TSC complex electrode was 18.5%) In the final stage, the largest weight loss, 32% for pure PPy and 36% for the PPy‐TSC complex observed between 350 and 800 °C, is attributed to the degradation and interchain crosslinking of the polymer. This result showed that the TSC complex doping added to the PPy structure slightly increases the thermal stability of the electrode but rather affects its morphological properties and accordingly its energy storage behavior.

As a result of the contribution of TSC complex to the polypyrrole conductive polymer, its porosity, and specific surface areas were investigated by nitrogen adsorption‐desorption isotherms and BET point plot (Figure 5c,d). The electrodes' surface area values were 8.5799 and 33.319 m^2^ g^−1^, respectively, obtained from BET for PPy and PPy‐TSC complex electrodes. PPy‐TSC complex's pore volume was determined to be 0.129 cg, while the pore volume of the PPy electrode was 0.048 cg according to the adsorption isotherms. The surface area and pore volume of supercapacitor electrode materials are the most important parameters in energy storage. It ensures that the electrode leaking through the pore volumes to the electrode surface has the opportunity to interact with the electrode.^[^ ^3^ ^]^

**Figure 6:**
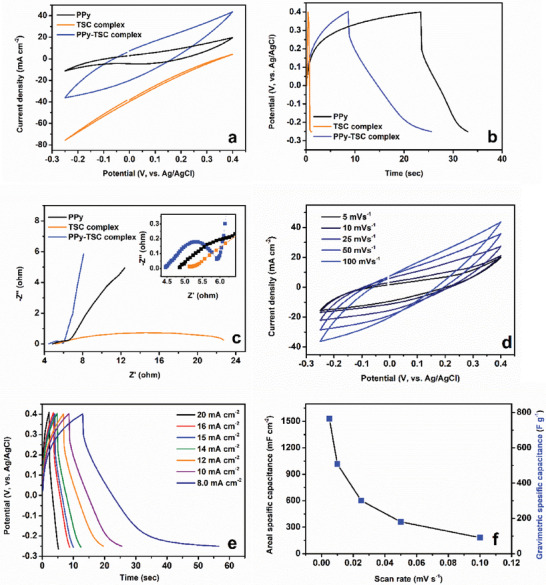
a) CV at 100 mV s^−1^ b) GCD at 10 mA cm^−2^, c) Nyquist plots at 1–100 000 Hz, d) Cyclic voltammograms of PPy‐TSC complex at different scan rates, e) GCD curves of PPy‐TSC complex at different current densities, f) Specific capacitance values from CVs.

The supercapacitor performance of the electrodes coated on the carbon‐felt was investigated. CV, GCD, and Nyquist analyses were performed to investigate the energy storage properties of the combination of TSC complex, polypyrrole, and both electrode materials in **Figure** **6**a–c.

Electrodes were optimized in the range of −0.25 V−(0.4) V versus Ag/AgCl potential window. The PPy‐TSC complex electrode showed the largest integration area at 100 mV s^−1^ in Figure 6a. The TSC complex alone did not exhibit super capacitive behavior, but when combined with PPy, it enhanced the capacitive performance. The specific capacitance for PPy, TSC complex, and PPy‐TSC complex electrodes were 31.7 (63.4 mF cm^−2^), 10.4 (10.4 mF cm^−2^) and 91.0 (182.0 mF cm^−2^), F g^−1^, respectively, at 100 mV s^−1^ scan rate. As a result of the CV measurements, the specific capacitance of the PPy‐TSC complex material increased by approximately three times compared to pure polypyrrole. The possible redox contribution of PPy to energy comes from its ability to undergo reversible oxidation and reduction during charge‐discharge cycles, contributing to the overall capacitance through faradaic reactions. Polypyrrole is an electrochemically active material that stores energy through redox reactions occurring on its surface. These reactions enhance the charge‐carrying capacity of polypyrrole. During oxidation, polypyrrole loses electrons, while it gains electrons during the reduction phase. This redox cycle facilitates charge transfer during the energy storage and release phases. The Faradaic charge‐transfer reaction process at the device can be delineated as follows:

(5) $$PPy^{n+}\cdot nA^{-}+ne^{-}↔PPy+nA^{-}$$

PPyn+ denotes the electrochemically positively doped polypyrrole electrode, whereas A—represents the dopant anion utilized in the electrochemical process. Upon the application of an external voltage to a pseudo‐capacitor, a reversible redox reaction transpires, facilitating charge transfer between the electrode surface and the electrolyte.^[^ ^18^, ^57^, ^58^ ^]^

When the surface properties were compared morphologically, it was observed that the PPy‐TSC complex electrode consists of small particles in a nano‐worm structure. This increases the interaction of the electrode with the electrolyte, which allows easy ion transfer to the electrode surface. The TSC complex, which is connected to the polymer structure through ionic and coordinative covalent bonds via pyrrole, has a positive effect on this behavior due to the Fe ion in its structure and the ability to perform electron delocalization. In addition, the presence of highly electronegative N, Cl, and Br groups in the complex enabled the complex to attract more electrons. In addition, the fact that the compound is a multistable ion, such as the center atom, allows it to perform more than one redox reaction. GCD tests of electrodes were performed at 10 mA cm^−2^ in the operating potential window range of −0.25 V– (0.4) V (in Figure 6b).

The values were calculated according to Equation (2), 76.9 (153.8 mF cm^−2^), 5.2 (10.5 mF cm^−2^), and 126.7 (253.4 mF cm^−2^) F g^−1^. The PPy‐TSC complex electrode exhibited the best charge storage performance. As the electrolyte/electrode surface interaction increased, the increased electrolyte ions on the surface provided the longest discharge time. As observed in the SEM images, the TSC complex rendered the polypyrrole surface significantly rougher. The formation of smaller‐sized material increased the surface area, thereby enhancing the capacitive performance.

EIS was performed to explain the behavior of the electrode materials over the electrolyte resistance (Rs) of the electrode materials coated on the carbon felt and the charge transfer resistance (Rct) on the interface between electrode/electrolyte.^[^ ^59^ ^]^ The EIS tests were made at 1–100 000 Hz with an amplitude of 5.0 mV and were presented in Figure 6c. Rs occurs due to electronic and ionic effects, while interfacial resistance refers (Rct) to the resistance between the active capacitive material and carbon felt substrate. Rs relates to the behavior of ions moving through the pores by diffusion.^[^ ^60^ ^]^ The values of Rs and Rct of PPy, TSC‐complex, and, PPy‐TSC complex electrode materials are presented in **Table** **1**. These results showed that the PPy‐TSC complex electrode material exhibited a low Rs and a low Rct. The electrode and the electrolyte can easily transfer charges due to the low resistance. The facilitation of ion transfer enhances the capacitance value.

**Table 1:** The value of the electrode active materials related to charge‐transfer resistance (Rct) and electrolyte resistance (Rs).

Electrode	Electrolyte resistance [Rs] [Ω]	Charge–transfer resistance [Rct] [Ω]
PPy	4.86	1.56
TSC complex	5.14	17.61
PPy‐TSC complex	4.47	1.45

According to Table 1, the PPy‐TSC complex had lower recorded Rs and Rct values than the other electrode materials, at 4.47 and 1.56 Ω, respectively. One of the main factors contributing to the PPy‐TSC complex's superior electrochemical performance and higher specific capacitance when compared to other electrode‐active materials is its low Rs and Rct. The impedance spectroscopic examination of the electrode material obtained with the TSC complex added during polypyrrole synthesis is in good agreement with galvanostatic charge/discharge and cyclic voltammetric studies.

Figure 6d shows cyclic voltammetry of the PPy‐TSC complex electrode from 5 to 100 mV s^−1^ in −0.25 V‐(0.4) V. The cyclic voltammogram curves increased gradually with the scan rate, indicating that the current was related to the scan rate. The cyclic voltammogram of the produced electrode material showed similarity to the voltammogram pattern of polypyrrole‐based materials.^[^ ^61^, ^62^, ^63^ ^]^ While pseudocapacitive behavior was observed more clearly at scan rates of 5 and 10 mV s^−1^, at higher scan rates the material behavior was not reflected in the voltammogram because the oxidation and reduction processes could not reach the scan rate.

Figure 6e shows the charge–discharge curves ranging from 8.0 to 20.0 mA cm^−2^. At 8.0 mA cm^−2^, the maximum Cs was calculated to be 264.9 F g^−1^ (529.8 mF cm^−2^). In Figure 6f, areal and gravimetric capacitances obtained from the cyclic voltammetry measurements taken in Figure 6d are presented. The electrode material exhibited the highest capacitance value of 764.6 F g^−1^ at 5 mV s^−1^.

**Figure 7:**
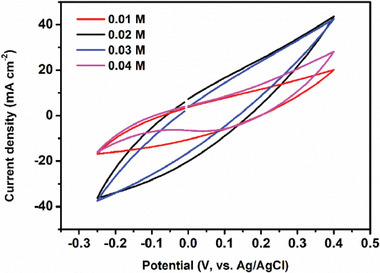
Concentration optimization of 2‐hiydroxy‐5‐bromo‐benzaldehyde‐S‐metyl on PPy polymerization.

Optimization of the concentration of TSC complex on the electrode material was performed for their energy storage performance. (**Figure** **7**). Here, the highest result was obtained with 0.02 m TSC complex. At values higher than this concentration, the TSC complex started to recrystallize, which had a negative effect on the electrode material, affecting homogeneity.

**Figure 8:**
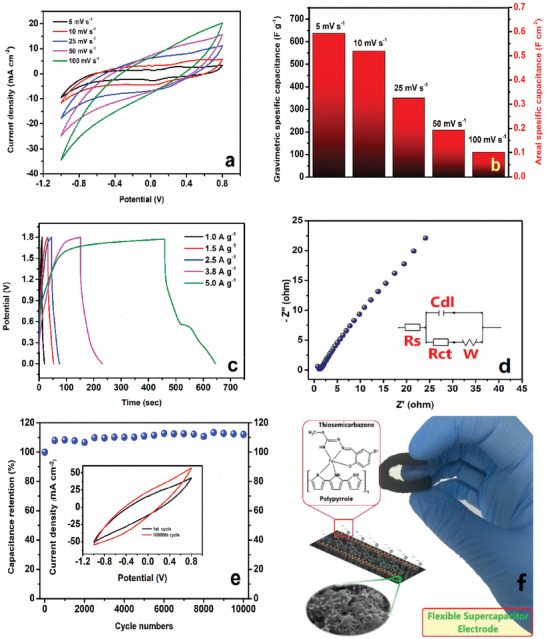
a) CVs of the asymmetric supercapacitor, b) Gravimetric and areal Cs obtained from CVs, c) GCD test at different current densities, d) Nyquist plot and its equivalent circuit model, e) Long‐life test at 0.5 Vs^−1^, f) Illustration of supercapacitor electrode.

The PPy‐TSC complex electrode and graphite electrode were asymmetrically combined, and their capacitance performance in a two‐electrode system was tested (**Figure** **8**a–e). Figure S2 (Supporting Information) shows the photo where the ASC module lights a red LED during bending and twisting. The equivalent circuit is shown in Figure S2b (Supporting Information). A resistive network is conducted to achieve the current limiting of ASC.^[^ ^64^ ^]^ In addition, LED performance video is presented in the Video S1 (Supporting Information). Figure 8a shows the CV of ASC at different scan rates The supercapacitor reached a potential of up to 1.8 V. Aqueous electrolytes are limited by the thermodynamic dissociation voltage of water, which is 1.23 V. Water does not dissociate at 1.23 V for two primary reasons. The overpotential necessary for hydrogen and oxygen evolution varies for each electrode surface. Second, the presence of impact ions in electrolytes complicates the dissociation process of water.^[^ ^65^ ^]^ The utilization of carbon‐based materials as current collectors, particularly in aqueous electrolyte systems, enhances the operating potential range by elevating the water dissociation potential.

The PPy‐TSC complex electrode showed pseudocapacitor behavior in the range of positive and negative current regions according to cyclic voltammetry as a result of the redox activity due to polypyrrole and TSC‐iron complex.^[^ ^66^ ^]^ The device gave a pair of redox peaks at potential points of 0.04 and 0.28 V at 5 mV s^−1^. It was observed that the peaks broadened with the effect of the scan rate. Figure 8b was presented Cs values from 5 to 100 mV s^−1^. The supercapacitor capacitance values ranged from 636.2 to 109.4 mF cm^−2^ (gravimetric capacitance 318.1 and 54.7 F g^−1^)5 to 100 mV s^−1^, respectively.

The charge‐discharge behavior of the ASC was investigated between 1.0 and 5.0 A g^−1^(Figure 8c). Depending on the internal resistance of ion diffusion at the felt electrode and the resistance of the electrolytes, a decrease in IR was observed. The discharge curves showed that at a current density of 1.0 A g^−1^, the IR drop was ≈0.2 V. The highest Cs obtained was calculated to be 62.8 F g^−1^. Some PPy‐based supercapacitor electrode materials and their devices were reported in Table S1 (Supporting Information).

The conductivity results of the electrode material produced for ASC using four probe methods are presented in **Table** **2** and Figure S5 (Supporting Information).

**Table 2:** The conductivity and resistivity values of the PPy‐TSC Complex Electrode Material.

Temperature [°C]	Resistivity [Ω × cm]	Electrical conductivity [S cm^−1^]
27	1.15	0.87
48	1.13	0.88
70	1.35	0.74
96	1.45	0.69
73	1.54	0.65
50	1.66	0.60

The four‐point probe method was utilized to characterize the electrical properties of the PPy‐TSC Complex electrode material, and the measurements were taken in a vacuum and inert environment, depending on the temperature. The measurement was carried out utilizing a small section of the prepared electrode. The conductivity value was calculated as 0.87 S cm^−1^ at room temperature, while an increase in resistance was observed with the rise in temperature.

The equivalent circuit model for fitting to the test of electrochemical impedance spectroscopy was shown in Figure 8d. The electrochemical properties were determined using a Nyquist plot for the asymmetric supercapacitor within 0.1 Hz–100 000 Hz at a 5 mV amplitude was shown.^[^ ^4^, ^10^ ^]^ Rs, the capacitance Cdl (parallel to Rct), and a Warburg diffusion (W) component attributed to ion diffusion are all components of the equivalent circuit.^[^ ^59^ ^]^ Rs was determined to be 6.69 Ω. Rct, W, and C_dl_ were calculated at 4.74 Ω, 1.81 e^1^ and 1.36e^−3^, respectively. Its super capacitance behavior was demonstrated by the results of the cyclic voltammetric investigation and the impedance spectroscopy analysis of the device. It was seen that the electrode material exhibits pseudocapacitance‐type reactive charge storage throughout its volume. Redox faradaic reactions and charge storage processes are facilitated at the electrode‐electrolyte interface by the diffusion of electrolyte ions into the electrode material.

More significantly, Figure 8e depiction of the energy storage device's capacitance retention was necessary for any realistic supercapacitor application A long cycle test of 10 000 cycles for capacitance retention was conducted, with a potential range of 1.8 V and a scan rate of 0.5 V s⁻¹. The device's Cs were retained even after 10 000 cycles.  After a certain number of cycles, the adsorption and desorption of electrolyte ions from the surface of the ASC with a capacitance protection value of 112% may be maximized. Despite variations in the number of cycles, a stable capacitance value was generally maintained over 10 000 cycles. Generally, conducting polymers for supercapacitors have a rather weak cycle stability dependence, since the redox points in the polymeric matrix are not steady enough and the polymer (backbone) can be damaged after long charge/discharge cycles.^[^ ^67^, ^68^, ^69^ ^]^ The redox sites of the polypyrrole‐based supercapacitor electrode material synthesized with the TSC complex exhibited stable behavior, successfully passing the test and supporting its long‐term use.

**Figure 9:**
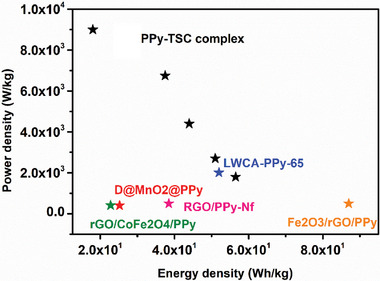
Ragone plot of comparison PPy‐based supercapacitors.

Energy and power density values calculated according to Equations (3) and (4) were in the range of 56.5–18.0 Wh kg^−1^ for the supercapacitor and power density values in the range of 1800–9000 W kg^−1^. It was compared with some PPy‐based supercapacitor applications in the literature and presented in the Ragone plot in **Figure** **9**. D@MnO2@PPy//D@FeOOH@PPy asymmetric device (25.2 Wh kg^−1^ at 400.0 W kg^−1^),^[^ ^70^ ^]^ LWCA‐PPy‐65 device(52.0 Wh kg^−1^ at 2012.3 W kg^−1^),^[^ ^71^ ^]^ Fe2O3/rGO/PPy device 87Wh kg^−1^ at 500 W kg^−1^),^[^ ^72^ ^]^ rGO/CoFe_2_O_4_/PPy device (22.8 Wh kg^−1^ at 410 W kg^−1^),^[^ ^73^ ^]^ RGO/PPy‐Nf device (38.5 Wh kg^−1^ at 500 W kg^−1^).^[^ ^74^ ^]^

## 4 Conclusion

In this work, a transition metal complex of iron (III) chloride and 2‐hydroxy‐5‐bromobenzaldehyde thiosemicarbazone was synthesized as a starting material. Then, an electrode material was synthesized by solvothermal reaction of polypyrrole and the TSC complex on a carbon‐felt electrode. The characterization of the complex and electrodes was carried out using UV–vis, elemental analysis, FT‐IR, XRD, BET, and TGA methods, and the surface morphology was examined by SEM technique. The PPy‐TSC complex electrode had three times more specific capacitance value compared to pure polypyrrole. After binding the thiosemicarbazone complex to the electrode structure, it was observed that the structure with cauliflower‐like morphology was transformed into a nano‐worm structure. The nano‐worm morphology of the electrode material containing 0.02 m TSC complex enhanced the interaction with the electrolyte and improved the capacitive properties of the electrode. The formed structure is suggested to increase the energy storage behavior by increasing the surface area and pore volume. The electrode material reached a value of 764.6 F g^−1^ at 5 mV s^−1.^ Moreover, the asymmetric supercapacitor exhibited a large operating potential of 1.8 V, allowing it to reach higher energy density values (56.5 Wh kg^−1^ at 1.0 Ag^−1^). At the end of the long cycle test, it was observed that it maintained its capacitance and even increased it slightly by reaching equilibrium and maintained this stability up to 10 000 cycles.

## Conflict of Interest

The authors declare no conflict of interest.

## Supporting information



Supporting Information

Supplemental Video 1

## Data Availability

The data that support the findings will be available in [Patent] at [URL/DOI] following an embargo from the date of publication to allow for commercialization of research findings.
