# Supplementary material for: A Flexible Asymmetric Supercapacitor with High‐Performance and Long‐Lifetime: Fabrication of Nanoworm‐Like‐Structured Electrodes Based on Polypyrrole‐Thiosemicarbazone Complex
Source: Small Methods. 2024 Oct 30;9(3):2401140. doi: 10.1002/smtd.202401140 (PMC11926503; doi:10.1002/smtd.202401140)
Supplement: Supplementary file 1 — Supporting Information [file SMTD-9-2401140-s002.docx]

**Supporting Information**

**A Flexible Asymmetric Supercapacitor with High-Performance and Long-Lifetime: Fabrication of Nanoworm-Like-Structured Electrodes Based on Polypyrrole-Thiosemicarbazone Complex**

Elif AVCU ALTIPARMAK ^1^**,** Sibel YAZAR ^2,^***,** Tulay BAL-DEMIRCI ^1,^*

^1^ Department of Chemistry, Engineering Faculty, Inorganic Chemistry Department, Istanbul University-Cerrahpasa, 34320 Istanbul, Turkey

^2^ Department of Chemistry, Engineering Faculty, Department of Physical Chemistry, Istanbul University-Cerrahpasa, 34320 Istanbul, Turkey

Addresses correspondence to E-mail: tulaybal@iuc.edu.tr, sibelyazar@iuc.edu.tr


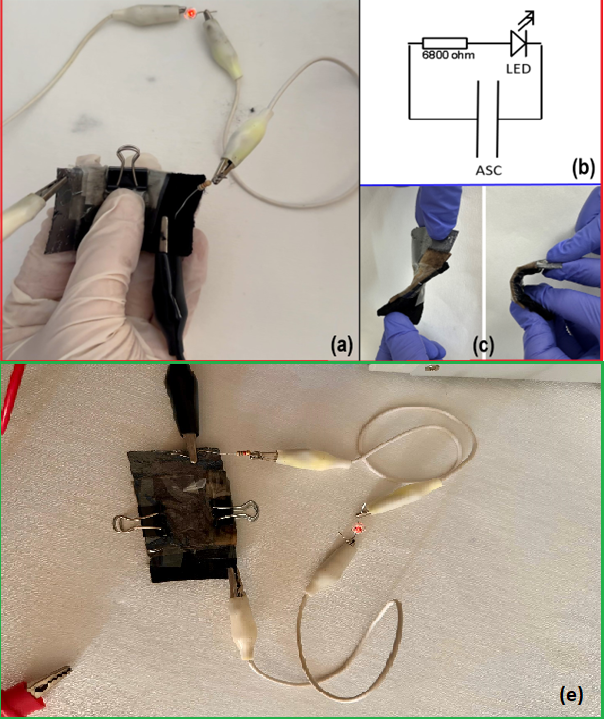


ASC

**Figure S1.** Photo of ASC a),e) Module lighting a red LED, b) the equivalent circuit and, c) during bending and twisting,


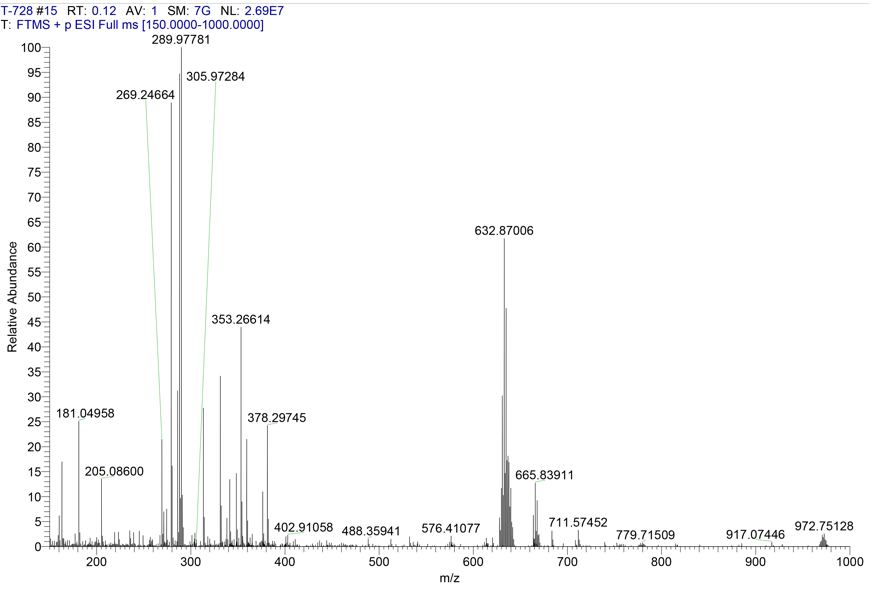


**Figure S2.** HRMS Spectrum of the TSC Complex.


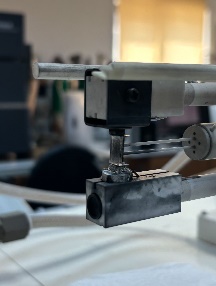

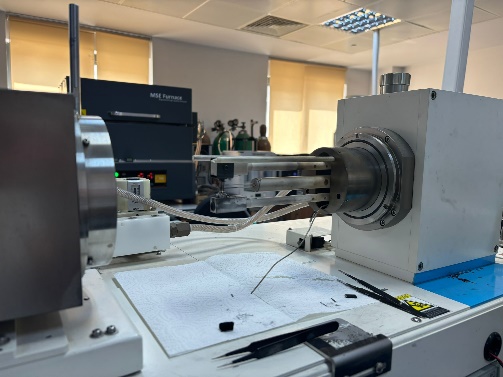

**Figure S3.** Temperature-Dependent Conductivity Graph of PPy-TSC Complex Electrode Material.

*
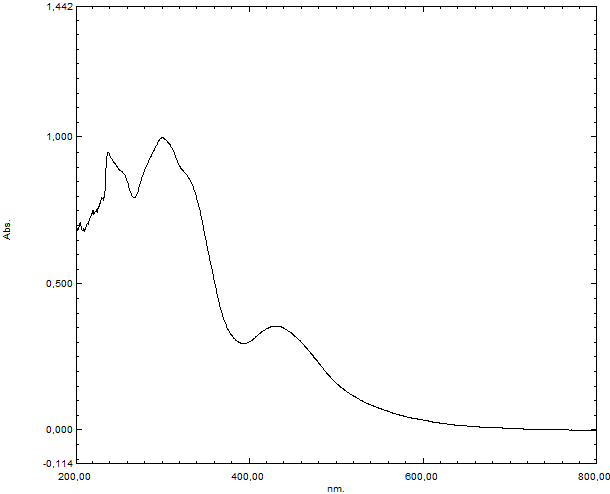
*

**Figure S4.** UV-Vis Spectrum of the TSC Complex. (10^-5^ M, CHCl_3_)





**Figure S5.** FTIR spectra of the Carbon Felt, Carbon Felt/PPy, Carbon Felt/PPy-TSC Complex.


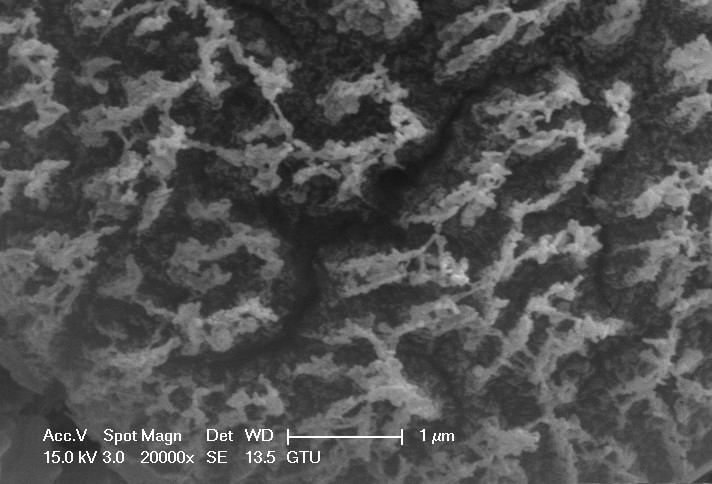


**Figure S6.** SEM image of TSC Complex.


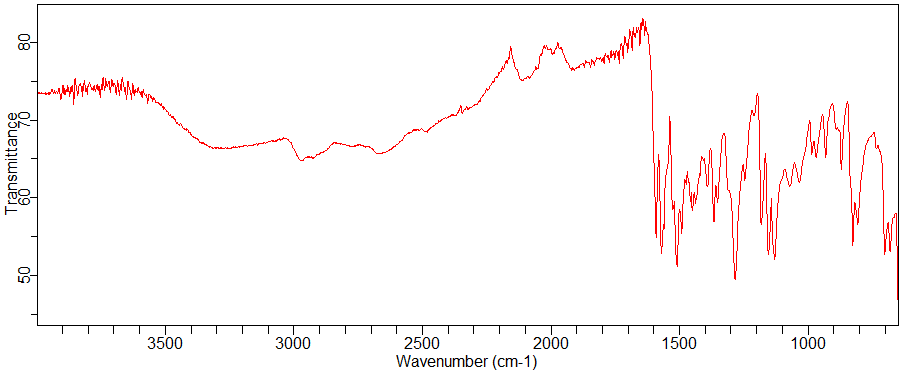


**Figure S7**. IR Spectrum of TSC complex.

**Table S1:** The conductivity and resistivity values of the PPy-TSC Complex Electrode Material.

| **Temperature (°C)** | **Resistivity (ohm*cm)** | **Electrical Conductivity (S/cm)** |
| --- | --- | --- |
| 27 | 1.15 | 0.87 |
| 48 | 1.13 | 0.88 |
| 70 | 1.35 | 0.74 |
| 96 | 1.45 | 0.69 |
| 73 | 1.54 | 0.65 |
| 50 | 1.66 | 0.60 |

**Table S2**. The list of the PPy-based electrodes and devices

| **Electrode materials** | **Electrolyte** | **Measurement type** | **Max. Capacitance** | **Cycling** | **Ref.** |
| --- | --- | --- | --- | --- | --- |
| PPy/HNB | 1.0 M H_2_SO_4_ | 3 -electrode  2 -electrode | 108.0Fg^-1^**,** 0.4 Ag^-1^  182Fg^-1^, 5.0 mVs^-1^ | 5,000 (91%) | [47] |
| LWCA-PPy-65 | 6.0 M KOH  6.0 M KOH  1.0 M H_2_SO_4_ | 3 -electrode  2 -electrode  2 -electrode | 421.5F g^-1^, 0.5 Ag^-1^  374.1Fg^-1^, 1.0 Ag^-1^  - | 5,000 (83%)  5,000 (90%)  5,000 (93%) | [48] |
| WO_3_-WS_2_/PPy | 1.0 M Na_2_SO_4_ | 3 -electrode | 624Fg^-1^, 3.0 Ag^-1^ | 5,000 (81%) | [49] |
| PPy/CNC-COO^−^-Cl^−^(ClO_4_^−^)_0.5 SC | 1.0 M KCI | 2 -electrode | 183.4Fg^−1,^ 0.2Ag^-1^ | 5,000 (103%) | [50] |
| Fe_2_O_3_@PPy//MnO_2_ SC | 0.5 M Na_2_SO_4_ | 3 -electrode | 1167.8F g^-1^, 1.0 Ag^-1^ | 3,000 (97%) | [51] |
| PPy/carbon-coated heat-treated carbon | 1.0 M KCl | 3 -electrode | 31.09F g^-1^, 1.0 Ag^-1^ | 3,000 (77 %) | [52] |
| PPy /ACFF | 2 M H2SO4 | 3 -electrode | 302F g^-1^, 0.01 Ag^-1^ | 3,000 (95%) | [53] |
| MoS_2_-PPy | 1 M KCI | 3 -electrode | 130Fg^-1^, 1.0Ag^-1^ | 2,000 (82%) | [37] |
| PPy-TSC complex | 1.0 M KCl | 3 -electrode  2 -electrode | 764.6Fg^-1^**,** 5.0mV s^-1^  318.1Fg^-1^**,** 5.0mV s^-1^ | 10,000  (112 %) | [This work] |


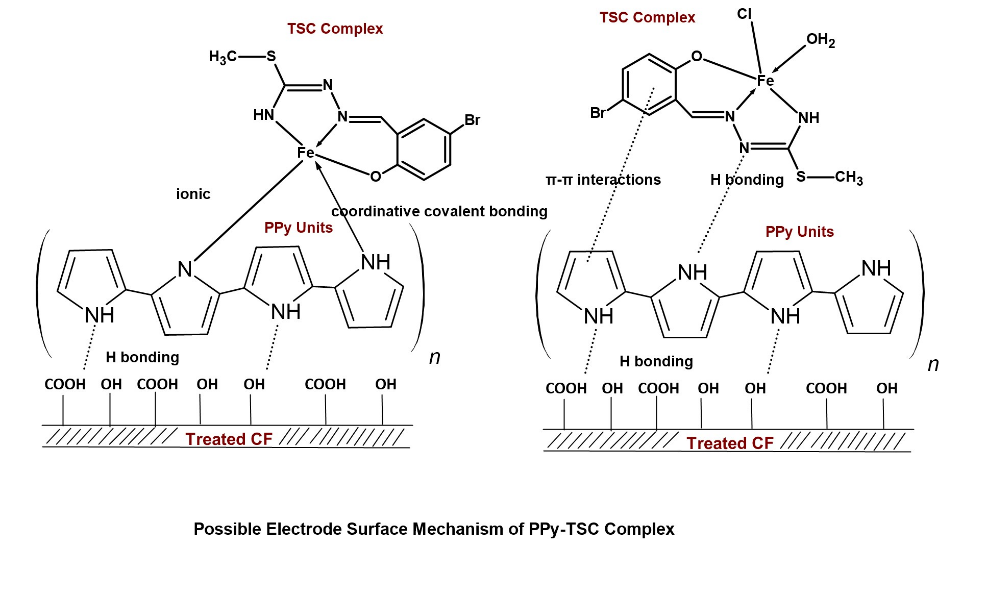


**Scheme S1**. Possible electrode surface mechanism of PPy-TSC Complex on carbon felt
